# Supplementary material for: JAK/STAT controls organ size and fate specification by regulating morphogen production and signalling
Source: Nat Commun. 2017 Jan 3;8:13815. doi: 10.1038/ncomms13815 (PMC5216089; doi:10.1038/ncomms13815)
Supplement: Supplementary Information — Supplementary Figures, Supplementary Table. [file ncomms13815-s1.pdf]

Supplementary Information

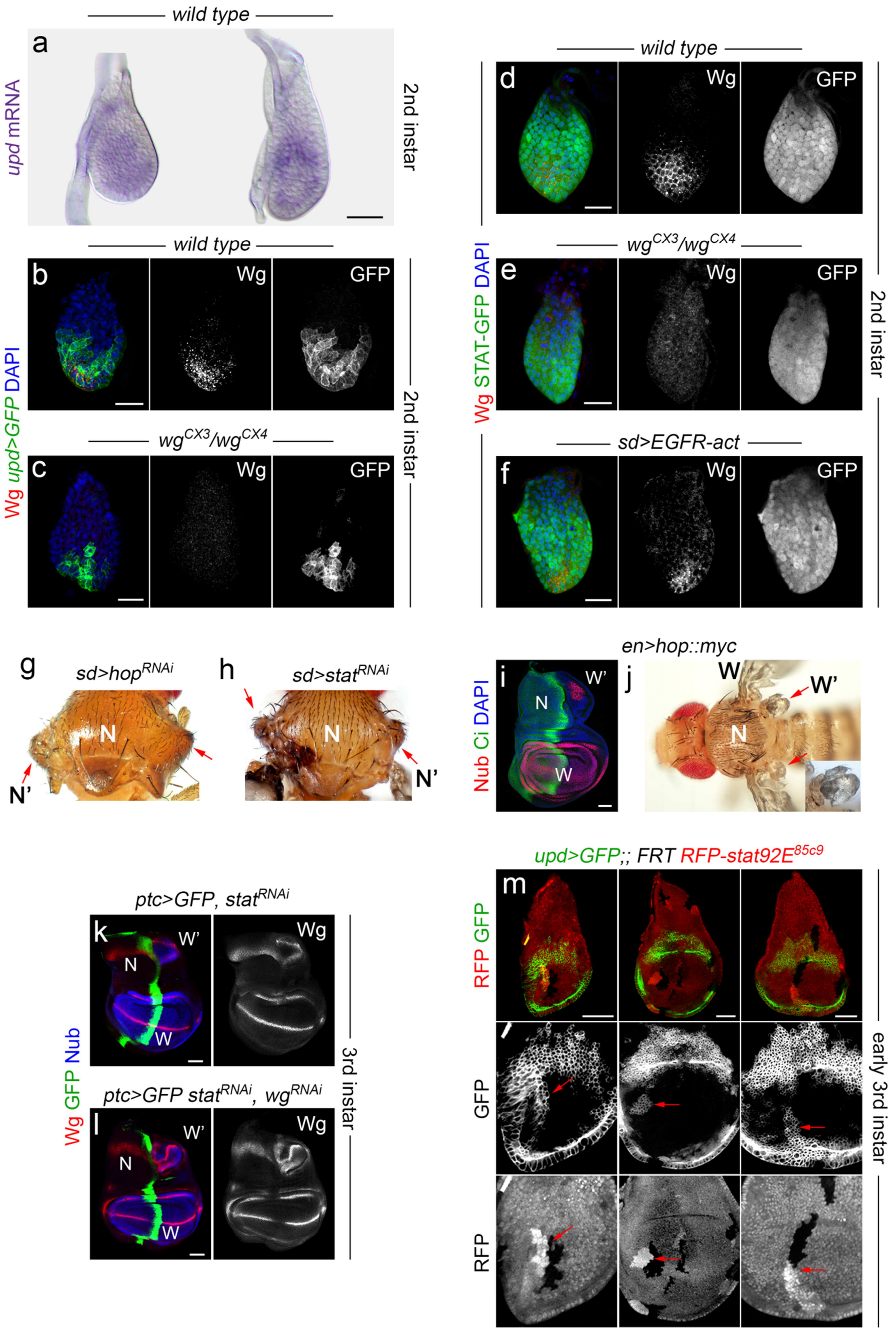

Supplementary Figure 1. JAK/STAT in early wing development

(**a**-**f**) Wing primordia of second instar larvae of the indicated genotypes labeled to visualize expression of *upd* mRNA (purple, **a**), Wingless protein (Wg, red or white, **b**-**f**), Unpaired (green or white, **b**, **c**, in *upd-gal4*, *UAS-myrGFP* larvae), activity of the JAK/STAT pathway (green or white, **d**-**f**, in *10xSTAT-GFP* larvae), and DAPI (blue, **b**-**f**). (**g**, **h**) Adult thoraxes of male individuals expressing the indicated transgenes under the control of the *sd-gal4* driver. Endogenous nota (N) and duplicated nota territories (N') are marked. Red arrows point to duplicated nota. (**i, j**) Late third instar wing primordium (**i**) or adult fly (**j**) expressing *hop::myc* under the control of the *en-gal4* driver. The wing primordium was labeled to visualize expression of Ci (green), Nub (red), and DAPI (blue). Endogenous wing (W), endogenous notum (N) and ectopic wing structures (W') are marked. Red arrows in **j** point to ectopic wings arising from the mesothorax. Higher magnification of an ectopic wing is shown in the inset. (**k**, **l**) Late third instar wing disc expressing the indicated transgenes under the control of the *ptc-gal4* driver and stained for Wg (red or white), GFP (green) and Nub (blue). Wing territory (W), the endogenous notum (N) and the ectopic wing territory (W') are marked. (**m**) Early third instar larval wing primordia with clones of cells lacking *stat92E* activity and marked by the presence of two copies of RFP. Note that clones induce ectopic expression of *upd* (*upd-gal4, UAS-myrGFP*, green or white, red arrows). Scale bars, 20 µm (**a**-**f**) and 50 µm (**i**, **k**, **l**, **m**).

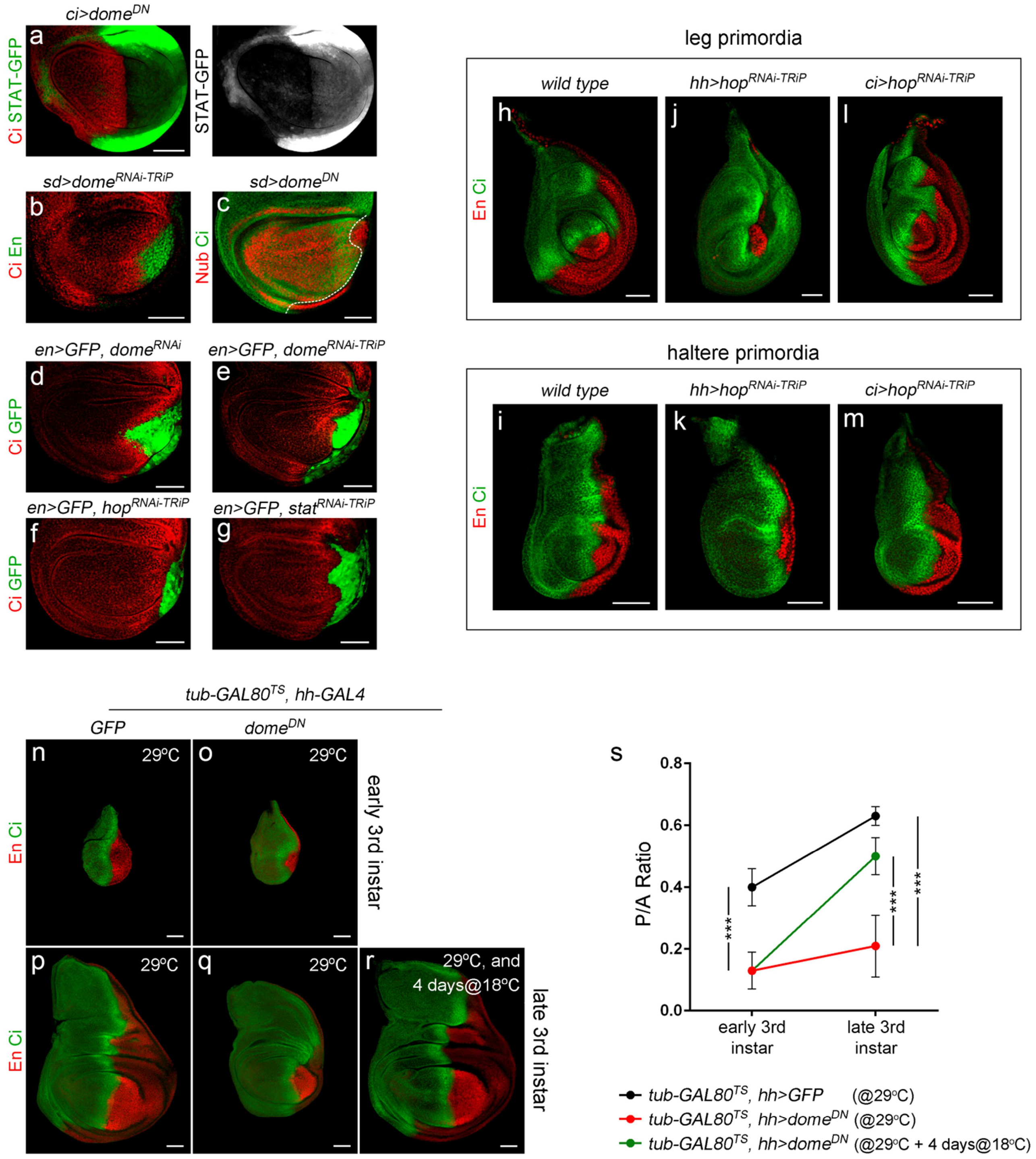

Supplementary Figure 2. JAK/STAT maintains the size of the P compartment

(**a**-**g**) Mature larval wing primordia expressing the indicated transgenes under the control of the *ci-gal4* (**a**), *sd-gal4* (**b**, **c**) or *en-gal4* (**d**-**g**) drivers and labeled to visualize activity of the JAK/STAT pathway (*10xSTAT-GFP*, green or white, **a**), expression of Ci (red, **a**, **b**, **d**-**g**, or green, **c**), En (green, **b**), Nub (red, **c**) and GFP (green, **d**-**g**). The white dashed line in **c** marks the AP boundary and the remaining P compartment maintains the wing identity monitored by Nub expression. (**h**-**m**) Mature larval leg (**h**, **j**, **l**) and haltere (**i**, **k**, **m**) discs from *wild type* larvae (**h**, **i**) or from larvae expressing hop^{RNAi} under the control of *hh-gal4* (**j**, **k**) or *ci-gal4* (**l**, **m**) labeled to visualize expression of Ci (green) and En (red). (**n**-**r**) Early (**n**, **o**) and late (**p**-**r**) third instar larval wing primordia expressing GFP (**n**, **p**) or $dome^{DN}$ (**o**, **q**, **r**) under the control of the *hh-gal4* driver and labeled to visualize expression of Ci (green) and En (red). Larvae were raised at 29 ºC until dissection (**n**-**q**) or raised

at 29 ºC and transferred to 18 ºC in early third instar and dissected in late third instar (**r**). The reduction in P size is largely restored after 4 days at the permissive temperature (18 ºC). Scale bars, 50 µm. (**s**) Graphic representation showing the average size of the P versus the A compartment (P/A ratio) of the wing primordia represented in **n**-**r**. P/A ratios: **n:** 0.4 ± 0.06 (n = 23); **o**: 0.13 ± 0.06 (n = 26); **p**: 0.63 ± 0.03 (n = 17); **q**: 0.21 ± 0.09 (n = 34); **r**: 0.5 ± 0.06 (n = 42). Error bars (vertical lines) represent standard deviation. *P*-values: Student's *t*-test, *** p<0.001.

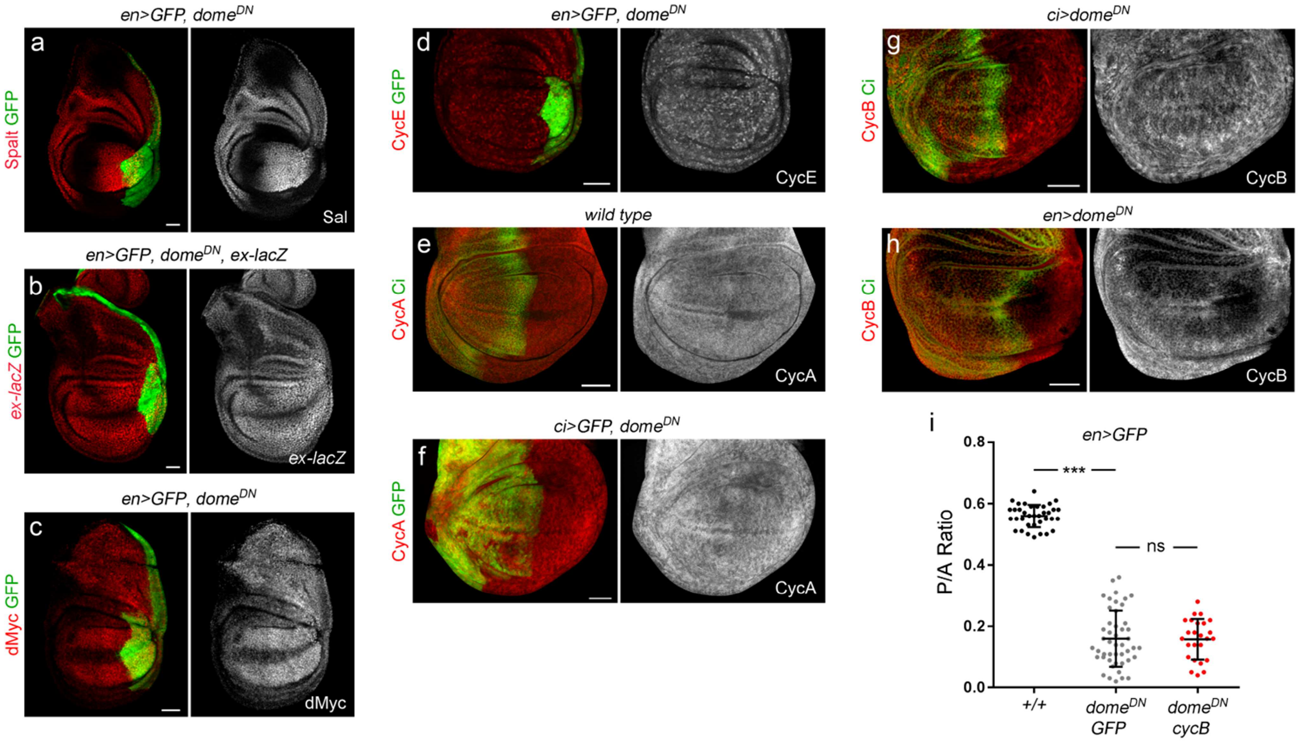

Supplementary Figure 3. JAK/STAT, growth promoting pathways and cell cycle regulation

(**a**-**h**) Mature larval wing primordia of the indicated genotypes labeled to visualize expression of GFP (green, **a**-**d**, **f**), Ci (green, **e**, **g**, **h**), Spalt (Sal, red or white, **a**), *expanded-lacZ* (antibody to β-gal, red or white, **b**), dMyc (red or white, **c**), CycE (red or white, **d**), CycA (red or white, **e**, **f**) and CycB (red or white, **g**, **h**). Scale bars, 50 µm. (**i**) Scatter plot showing the size of the P versus the A compartment (P/A ratio) of late third instar wing primordia expressing the indicated transgenes under the control of the *en-gal4* driver. P/A ratios from left to right: 0.56 ± 0.04 (n = 36); 0.16 ± 0.09 (n = 47); 0.16 ± 0.06 (n = 25). Error bars (vertical lines) represent standard deviation and long horizontal lines mark the average values. *P*-values: Student's *t*-test, ns = not significant; *** p<0.001.

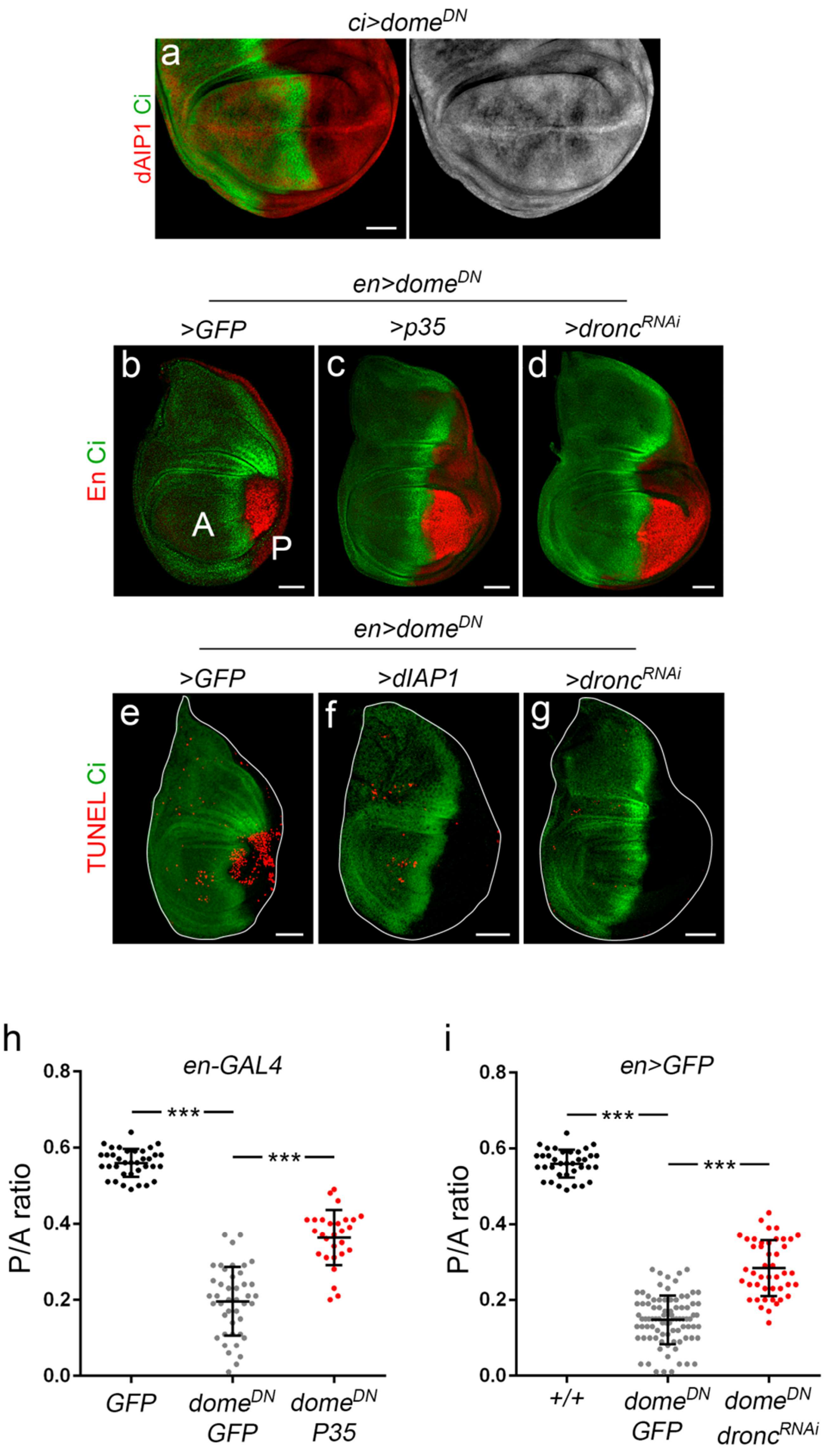

Supplementary Figure 4. JAK/STAT promotes survival of P cells through dIAP1

(**a**-**g**) Late third instar wing primordia from individuals of the indicated genotypes labeled to visualize expression of dIAP1 (red or white, **a**), En (red, **b**-**d**) and Ci (green) proteins, and apoptotic cells by TUNEL staining (red, **e**-**g**). The contour of the wing discs is marked by a white line in **e**-**g**. Scale bars, 50 µm. (**h**, **i**) Scatter plots showing the size of the P versus the A compartment (P/A ratio) of late third instar wing primordia expressing the indicated transgenes under the control of the *en-gal4* driver. In **h**, P/A ratios from left to right: 0.56 ± 0.04 (n = 36); 0.2 ± 0.09 (n = 43); 0.36 ± 0.07 (n = 28). In **i**, P/A ratios from left to right: 0.56 ± 0.04 (n = 36); 0.15 ± 0.06 (n = 87); 0.28 ± 0.07 (n = 45). Error bars (vertical lines) represent standard deviation and long horizontal lines mark the average values. *P*-values: Student's *t*-test, *** p<0.001.

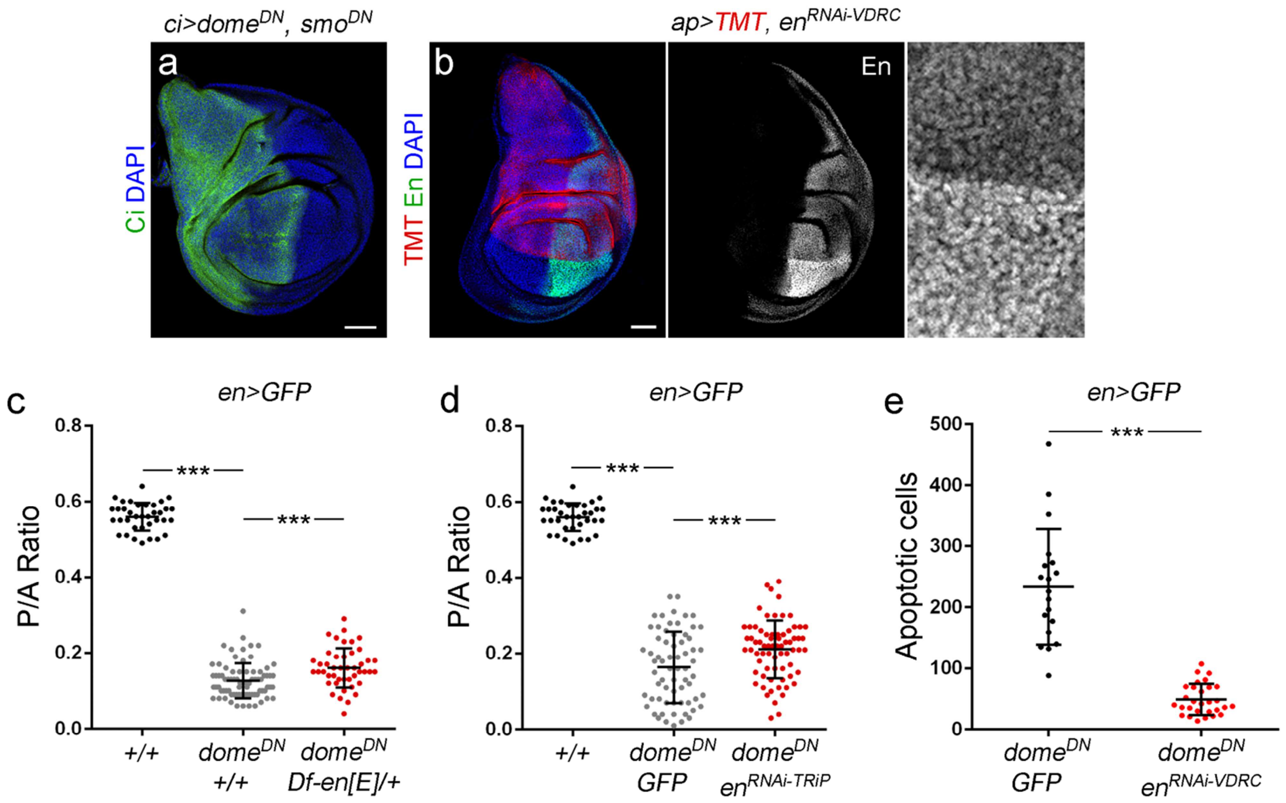

Supplementary Figure 5. JAK/STAT counteracts the effects of Engrailed on tissue growth

(**a**, **b**) Late third instar wing primordia from individuals of the indicated genotypes labeled to visualize expression of Ci (green, **a**), Engrailed (green, **b**), *Myristoylated-Tomato* (TMT, red, **b**) and DAPI (blue). Scale bars, 50 µm. (**c**, **d**) Scatter plots showing the size of the P versus the A compartment (P/A ratio) of late third instar wing primordia expressing the indicated transgenes under the control of the *en-gal4* driver. In **c**, P/A ratios from left to right: 0.56 ± 0.04 (n = 36); 0.13 ± 0.05 (n = 78); 0.16 ± 0.05 (n = 45). In **d**, P/A ratios from left to right: 0.56 ± 0.04 (n = 36); 0.16 ± 0.09 (n = 66); 0.21 ± 0.08 (n = 70). Error bars represent standard deviation. *P*-values: Student's *t*-test, *** p<0.001. (**e**) Scatter plot showing the absolute number of apoptotic cells in the P compartment of late third instar wing primordia from individuals of the indicated genotypes. Values from left to right: 233 ± 94 (n = 19); 49 ± 25 (n = 29). Error bars (vertical lines) represent standard deviation and long horizontal lines mark the average values. *P*-values: Student's *t*-test, *** p<0.001.

Supplementary Table

		Genotype
Figure 1	a	vn-gal4, UAS-GFP/+
	c	upd-gal4, UAS-myrGFP/+
	d	10xSTAT-GFP/+
	e, h, i	wild type
	f, j, k	sd-gal4/Y; dome$^{RNAi-VDRC}$/+; UAS-dcr2/+
	g	sd-gal4/Y; dome$^{RNAi-VDRC}$/+; UAS-dcr2/+,<br>sd-gal4/Y; UAS-dome$^{RNAi-VDRC}$/wg^{CX4}; UAS-dcr2/+,<br>sd-gal4/Y; UAS-dome$^{RNAi-VDRC}$/EGFR^{F2}; UAS-dcr2/+, and<br>sd-gal4/Y; UAS-dome$^{RNAi-VDRC}$/+; UAS-dcr2/iro^{EGP7}
Figure 2	a	ptc-gal4, UAS-GFP/UAS-sgg; tub-gal80^{TS}/+
	b	ptc-gal4, UAS-GFP/UAS-dome$^{RNAi-VDRC}$
	c, h	mirror-lacZ/+
	d, i, j	sd-gal4/Y; UAS-dome$^{RNAi-VDRC}$/+; UAS-dcr2/mirror-lacZ
	e, n	vn-lacZ/+
	f, g, o, p	sd-gal4/Y; UAS-dome$^{RNAi-VDRC}$/+; UAS-dcr2/vn-lacZ
	k	ap-lacZ/+
	l, m	sd-gal4/Y; UAS-dome$^{RNAi-VDRC}$/ap-lacZ; UAS-dcr2/+
Figure 3	a	ptc-gal4, UAS-GFP/+; mirror-lacZ/tub-gal80^{TS}
	b-f	ptc-gal4, UAS-GFP/UAS-upd; mirror-lacZ/tub-gal80^{TS}
	g	ptc-gal4, UAS-GFP/+; UAS-vn:aos/+
	h	10xSTATGFP/+
	i	ci-gal4, UAS-myrTomato, 10xSTAT-GFP/UAS-dome$^{RNAi-VDRC}$
	j	ci-gal4, UAS-GFP/UAS-stat92E$^{RNAi-VDRC}$; mirror-lacZ/+
	k	ci-gal4, UAS-GFP/UAS-dome$^{RNAi-VDRC}$
	l	ci-gal4, UAS-GFP/+; UAS-dome^{DN}/+
	m	ci-gal4/UAS-dome$^{RNAi-VDRC}$; UAS-dome^{DN}/+
	o	wild type and ci-gal4, UAS-GFP/UAS-dome$^{RNAi-VDRC}$
Figure 4	a	wild type
	b, c	sd-gal4/+;; UAS-dome^{DN}/+
	d	hop^{27}/Y
	e	en-gal4, UAS-GFP/+; UAS-dome^{DN}/+
	f	hs-FLP/+;; FRT82B/FRT82B M(3)95A2 UbiGFP
	g	hs-FLP/+;; FRT82B stat92E^{85c9} /FRT82B M(3)95A2 UbiGFP
	h	hs-FLP/+;; FRT82B/FRT82B M(3)95A2 UbiGFP, and<br>hs-FLP/+;; FRT82B stat92E^{85c9} /FRT82B M(3)95A2 UbiGFP
	j	hs-FLP/+;; FRT82B stat92E^{85c9} /FRT82B arm-lacZ
	k	UAS-FLP/ubi-FRT-stop-FRT-GFP; UAS-dome^{DN}/hh-GAL4
Figure 5	a-c	hop^{27}/Y
	d, f	wild type
	e	diap1-lacZ/+

**Figure 5**	**g**	*en-gal4, UAS-GFP/+; UAS-hop::myc/diap1-lacZ*
	**h**	*en-gal4, UAS-GFP/+; UAS-hop*$^{RNAi-TRiP}$*/diap1-lacZ*
	**i**	*en-gal4, UAS-GFP/+; UAS-hop*$^{RNAi-TRiP}$*/+*
	**j**	*en-gal4, UAS-GFP/UAS-GFP; UAS-dome*^{DN}*/+*
	**k**	*en-gal4, UAS-GFP/UAS-dIAP1; UAS-dome*^{DN}*/+*
	**l**	*en-gal4, UAS-GFP/+*<br>*en-gal4, UAS-GFP/UAS-GFP; UAS-dome*^{DN}*/+* and<br>*en-gal4, UAS-GFP/UAS-dIAP1; UAS-dome*^{DN}*/+*
	**m**	*en-gal4, UAS-GFP/+; UAS-dome*^{DN}*/+*
	**n**	*en-gal4, UAS-GFP/+*<br>*en-gal4, UAS-GFP/UAS-GFP; UAS-dome*^{DN}*/+,* and<br>*en-gal4>UAS-GFP/UAS-cycA; UAS-dome*^{DN}*/+*
**Figure 6**	**a, c**	*en-gal4, UAS-GFP/UAS-en*$^{RNAi-VDRC}$*; UAS-dome*^{DN}*/+*
	**b**	*en-gal4, UAS-GFP/UAS-GFP; UAS-dome*^{DN}*/+*
	**d**	*en-gal4, UAS-GFP/+*<br>*en-gal4, UAS-GFP/UAS-GFP; UAS-dome*^{DN}*/+,* and<br>*en-gal4, UAS-GFP/UAS-en*$^{RNAi-VDRC}$*; UAS-dome*^{DN}*/+*
	**e**	*en-gal4, UAS-GFP/UAS-RFP*
	**f**	*en-gal4, UAS-GFP/UAS-en*$^{RNAi-VDRC}$
	**g**	*en-gal4, UAS-GFP/UAS-RFP, and*<br>*en-gal4, UAS-GFP/UAS-en*$^{RNAi-VDRC}$
	**h-j, l**	*hh-gal4, tub-gal80*^{TS}*/UAS-en*
	**k**	*UAS-GFP/+; hh-gal4, tub-gal80*^{TS}*/+*
	**m**	*UAS-GFP/+; hh-gal4, tub-gal80*^{TS}*/+,* and<br>*hh-gal4, tub-gal80*^{TS}*/UAS-en*
**Figure 7**	**a**	*dpp-lacZ/+*
	**b, c**	*sd-gal4/+;; UAS-dome*^{DN}*/dpp-lacZ*
	**d**	*UAS-GFP/+; hh-gal4/UAS-dome*^{DN}
	**e**	*UAS-hh::GFP/+; hh-gal4/UAS-dome*^{DN}
	**f**	*UAS-GFP/+; hh-gal4/UAS-dome*^{DN}*,*<br>*UAS-hh::GFP/+; hh-gal4/UAS-dome*^{DN}*,* and<br>*UAS-dIAP1/+; hh-gal4/UAS-dome*^{DN}
**Figure 8**	**a**	*upd-gal4, UAS-myrGFP/+*
	**b**	*10xSTAT-GFP/+*
	**c, i**	*wild type*
	**d**	*ci-gal4, UAS-GFP/+; dome*^{DN}*/+*
	**e**	*dpp-lacZ/+*
	**f-h, j, k**	*hth-gal4, UAS-GFP/UAS-dome*^{DN}
**Figure S1**	**a**	*wild type*
	**b**	*upd-gal4, UAS-myrGFP/+*
	**c**	*upd-gal4, UAS-myrGFP/+; wg*^{CX3}*/wg*^{CX4}
	**d**	*10xSTAT-GFP/+*
	**e**	*wg*^{CX3}*/wg*^{CX4}*; 10XSTAT-GFP/+*
	**f**	*sd-gal4/UAS-EGFR*$^{\lambda TOP4.2}$*; 10XSTAT-GFP/+*

**Figure S1**	**g**	*sd-gal4/Y;; UAS-dcr2/UAS-hop*$^{RNAi\text{-}TRiP}$
	**h**	*sd-gal4/Y; UAS-stat92E*$^{RNAi\text{-}VDRC}$*/+; UAS-dcr2/+*
	**I, j**	*en-gal4, UAS-GFP/+; UAS-hop::myc/+*
	**k**	*ptc-gal4, UAS-GFP/UAS-stat92E*$^{RNAi\text{-}VDRC}$
	**l**	*ptc-gal4, UAS-GFP/UAS-stat92E*$^{RNAi\text{-}VDRC}$*; UAS-wg*$^{RNAi\text{-}TRiP}$*/+*
	**m**	*hs-FLP/upd-gal4, UAS-myrGFP;; FRT82B ubi-RFP, stat92E*^{85c9}*/FRT82B*
**Figure S2**	**a**	*ci-gal4, UAS-myrTomato, 10xSTAT-GFP; UAS-dome*^{DN}*/+*
	**b**	*sd-gal4/+;; UAS-dcr2/UAS-dome*$^{RNAi\text{-}TRiP}$
	**c**	*sd-gal4/+;; UAS-dome*^{DN}*/+*
	**d**	*UAS-dcr2/+; en-gal4, UAS-GFP/UAS-dome*$^{RNAi\text{-}VDRC}$
	**e**	*UAS-dcr2/+; en-gal4, UAS-GFP/+; UAS-dome*$^{RNAi\text{-}TRiP}$*/+*
	**f**	*UAS-dcr2/+; en-gal4, UAS-GFP/+; UAS-hop*$^{RNAi\text{-}TRiP}$*/+*
	**g**	*UAS-dcr2/+; en-gal4, UAS-GFP/+; UAS-stat92E*$^{RNAi\text{-}TRiP}$*/+*
	**h, i**	*UAS-GFP/UAS-GFP; hh-gal4/+*
	**j, k**	*UAS-GFP/+; hh-gal4/UAS-hop*$^{RNAi\text{-}TRiP}$
	**l, m**	*ci-gal4, UAS-GFP/+; UAS-hop*$^{RNAi\text{-}TRiP}$*/+*
	**n, p**	*UAS-GFP/+; hh-gal4, tub-gal80*^{TS}*/+*
	**o, q, r**	*hh-gal4, tub-gal80*^{TS}*/UAS-dome*^{DN}
	**s**	*UAS-GFP/+; hh-gal4, tub-gal80*^{TS}*/+*, and<br>*hh-gal4, tub-gal80*^{TS}*/UAS-dome*^{DN}
**Figure S3**	**a, c, d, h**	*en-gal4, UAS-GFP/+; UAS-dome*^{DN}*/+*
	**b**	*en-gal4, UAS-GFP/+; UAS-dome*^{DN}*/ex-lacZ*
	**e**	*wild type*
	**f, g**	*ci-gal4, UAS-GFP/+; UAS-dome*^{DN}*/+*
	**i**	*en-gal4, UAS-GFP/+*<br>*en-gal4, UAS-GFP/UAS-GFP; UAS-dome*^{DN}*/+*, and<br>*en-gal4, UAS-GFP/+; UAS-dome*^{DN}*/UAS-cycB:HA*
**Figure S4**	**a**	*ci-gal4, UAS-GFP/+; UAS-dome*^{DN}*/+*
	**b**	*en-gal4, UAS-GFP/+; UAS-dome*^{DN}*/+*
	**c**	*en-gal4, UAS-p35/+; UAS-dome*^{DN}*/+*
	**d, g**	*en-gal4, UAS-GFP/+; UAS-dome*^{DN}*/UAS-dronc*$^{RNAi\text{-}VDRC}$
	**e**	*en-gal4, UAS-GFP/UAS-GFP; UAS-dome*^{DN}*/+*
	**f**	*en-gal4, UAS-GFP/UAS-dIAP1; UAS-dome*^{DN}*/+*
	**h**	*en-gal4, UAS-GFP/+,*<br>*en-gal4, UAS-GFP/+; UAS-dome*^{DN}*/+*, and<br>*en-gal4, UAS-p35/+; UAS-dome*^{DN}*/+*
	**i**	*en-gal4, UAS-GFP/+*<br>*en-gal4, UAS-GFP/UAS-GFP; UAS-dome*^{DN}*/+* and<br>*en-gal4, UAS-GFP/+; UAS-dome*^{DN}*/UAS-dronc*$^{RNAi\text{-}VDRC}$
**Figure S5**	**a**	*ci-gal4/UAS-smo*^{DN}*; UAS-dome*^{DN}*/+*
	**b**	*ap-gal4, UAS-myrTomato/UAS-en*$^{RNAi\text{-}VDRC}$
	**c**	*en-gal4, UAS-GFP/+*<br>*en-gal4, UAS-GFP/+; UAS-dome*^{DN}*/+*, and

		en-gal4, UAS-GFP/Df(2)en^{E}; UAS-dome^{DN}/+
**Figure S5**	**d**	*en-gal4, UAS-GFP/+*<br>*en-gal4, UAS-GFP/UAS-GFP; UAS-dome^{DN}/+,* and<br>*en-gal4, UAS-GFP/+; UAS-dome^{DN}/UAS-en$^{RNAi\text{-}TRiP}$*
	**e**	*en-gal4, UAS-GFP/UAS-GFP; UAS-dome^{DN}/+,* and<br>*en-gal4, UAS-GFP/UAS-en$^{RNAi\text{-}VDRC}$; UAS-dome^{DN}/+*

Supplementary Table 1. List of fly genotypes
